# Leader perspectives on implementing Time Together: a qualitative process evaluation using the Consolidated Framework for Implementation Research

**DOI:** 10.1136/bmjopen-2026-117894

**Published:** 2026-06-24

**Authors:** Andreas Glantz, Britt-Marie Lindgren, Ingeborg Nilsson, Anna Westerlund, Ulla Hällgren Graneheim, Jenny Molin

**Affiliations:** 1 Department of Nursing, Umeå University, Umeå, Sweden; 2 Department of Community Medicine and Rehabilitation, Occupational Therapy, Umeå University, Umeå, Sweden; 3 Department of Epidemiology and Global Health, Umeå University, Umeå, Sweden; 4 Department of Health Sciences, University West, Trollhättan, Sweden; 5 Department of Clinical Sciences Psychiatry, Umeå University, Umeå, Sweden

**Keywords:** Implementation Science, QUALITATIVE RESEARCH, MENTAL HEALTH, Hospitals, Nursing Care, Quality Improvement

## Abstract

**Abstract:**

**Objectives:**

Time Together (TT) is a nursing intervention designed with a focus on increasing the possibility of patients and staff engaging in joint activities. Previous research points towards implementation difficulties but there are no studies specifically investigating the process of implementing TT in psychiatric inpatient care (PIC). Therefore, the aim of this study is to explore mid-level leaders’ and implementation leads’ perspectives on implementing TT in PIC, analysed through the Consolidated Framework for Implementation Research (CFIR).

**Design:**

This qualitative study used semistructured interviews with 11 mid-level leaders and implementation leads who took part in implementing TT. The interviews were analysed using deductive qualitative content analysis, with the CFIR applied as a structured matrix in the analysis.

**Setting:**

The study was conducted in PIC settings at three wards within secondary care healthcare services in northern Sweden.

**Participants:**

Participants were 11 mid-level leaders and implementation leads involved in the implementation of TT in PIC.

**Interventions:**

TT is scheduled for 1 hour, 5 days a week. During this hour, nursing staff and patients engage in joint activities, while one or two staff members oversee administrative duties. No meetings, visits or other ward-related activities are scheduled during this hour.

**Results:**

Deductive content analysis resulted in 15 categories sorted into 4 domains and 11 constructs. TT was perceived as advantageous and aligned with the wards’ mission. However, one key finding was that mid-level leaders and implementation leads found implementation challenging as they perceived that some innovation deliverers viewed joint activities within TT as lacking value and not being ‘proper work’. Furthermore, there was a need for more information and a clearer rationale for the intervention in order to improve implementation and delivery.

**Conclusions:**

The findings indicate a discrepancy in values and attitudes between leaders and innovation deliverers regarding what constitutes meaningful care in psychiatric inpatient settings. Implementation was further influenced by challenges related to leadership and motivating others. Barriers to implementing TT may not be addressable through implementation and intervention training alone. Instead, implementation planning should also consider intervention deliverers’ values and beliefs.

**STRENGTHS AND LIMITATIONS OF THIS STUDY**

- The study was guided by the Consolidated Framework for Implementation Research throughout both data collection and analysis, providing a coherent and theory-informed methodological approach.
- Deductive qualitative content analysis enabled a structured examination of implementation determinants while still allowing unanticipated constructs to be captured through comprehensive coding of the interview material.
- The use of a predefined analytical framework may have influenced how data were interpreted, and some findings could potentially be understood through multiple constructs.
- The study was conducted within a specific healthcare context, which may limit transferability to other settings or healthcare systems.

## Background

 International research focusing on psychiatric inpatient care (PIC) has for many years shown that there are challenges in offering care that is in line with what patients expect. A recent study on the opportunities for improvement in PIC showed that former patients had experienced a lack of empathy, communication and humane care while treated in PIC.^1^ Another often reported experience from patients within PIC is boredom, which may have consequences such as negative effects on mental health as well as longer therapy duration.^2 3^

In Sweden, the content of care within adult PIC is not regulated by law. The Swedish Association of Local Authorities and Regions however, state the importance of offering meaningful care. The care should include a variety of individualised activities and could include activities that are physical, relationship building and/or promoting social fellowship.^4^ This is also highlighted by patients as desirable but unfortunately often missing, with PIC characterised by loneliness and boredom.^5 6^ This is in line with studies highlighting that while nursing staff have a desire to spend time with patients,^7 8^ time is mostly spent on medication tasks or ward-related tasks.^9^

Time Together (TT) is a nursing intervention that was designed to increase the possibilities that joint activities with nursing staff and patients are offered in PIC.^10^ The intervention aims at allocating 1 hour, 5 days a week, when staff and patients join in activities. TT^10^,^12^ consists of the following four elements:

- TT is scheduled for 1 hour, Monday through Friday.
- The nursing staff engage with patients in joint activities during TT.
- During TT, one or two staff members oversee administrative duties.
- Meetings, visits or other ward-related activities are not scheduled during TT.

Joint activities in TT have a focus on the significance of the ordinary and are activities that promote the togetherness of patients and nursing staff and are commonplace in human lives. These activities can, for instance, be playing games, taking walks, watching movies or physical activities. The patient perspective is in focus, and activities are preferably chosen by the patients themselves.

There are several studies published in the last decade evaluating TT and the closely related intervention Protected Engagement Time (PET). Consistently, issues of introducing and implementing TT and PET in PIC have been raised.^10 13 14^ It has also proven difficult to properly evaluate TT and PET due to uncertainties regarding implementation and fidelity in delivery of the intervention. As early as 2014, barriers that might affect implementation of PET, including staff shortages and inconsistent implementation and delivery, were mentioned in a discussion paper.^15^

Within the field of implementation science, several theories, models and frameworks have been developed throughout the years with the aims to describe and/or guide the process of implementation and provide structure for evaluating implementation efforts. Nilsen^16^ categorised theoretical approaches in implementation science with *determinant frameworks* being one of the five identified categories. Determinant frameworks have a descriptive purpose, summarising determinants/factors, that often are clustered in domains, hypothesised to influence and explain implementation processes, outputs (ie, effects on healthcare practitioners and work practice) and outcomes (ie, effects on the target population).^16^ The Consolidated Framework for Implementation Research (CFIR) is a determinant framework based on a synthesis of constructs from previously existing theories, models and frameworks.^17^ The framework, which was updated in 2022,^18^ can, for instance, be used to identify and explain which barriers and facilitators impact an implementation effort both prospectively and retrospectively. CFIR consists of five major domains: the *innovation*, *inner* and *outer settings*, the *individuals* involved and the *implementation process*. Across these domains, the framework comprises 48 constructs and 19 subconstructs, such as the innovation’s relative advantage, structural characteristics and culture of the inner setting, policy, financing and attitudes of the outer setting, individuals such as mid-level leaders, opinion leaders and innovation deliverers and aspects of the implementation process, such as teaming, planning or tailoring strategies.^18^

While previous qualitative studies of TT have primarily explored experiences of the intervention in practice, there are no studies specifically investigating the process of implementing TT in PIC. Furthermore, no studies have to our knowledge used CFIR to investigate barriers and facilitators for implementing nursing interventions in PIC. By applying CFIR as an analytical lens, the present study focuses on identifying implementation determinants across domains such as the innovation, inner setting and individuals, thereby complementing previous qualitative research on TT. Leadership perspectives are crucial in the implementation of nursing interventions in PIC, as mid-level leaders and implementation leads play a central role in facilitating implementation in everyday practice. Therefore, the aim of this study is to explore mid-level leaders’ and implementation leads’ perspectives on implementing TT in PIC, analysed through CFIR.

## Methods

### Study design

This qualitative study used semistructured interviews with mid-level leaders and implementation leads to explore perspectives on implementing TT in PIC. A deductive approach was used, and both the interview guide and the analysis were guided by CFIR.^17 18^ Data were analysed using deductive content analysis.^19^ The study followed the consolidated criteria for reporting qualitative research.^20^

### Patient and public involvement

Patients and/or the public were not involved in the design, or conduct, or reporting, or dissemination plans of this research.

### Setting

Healthcare in Sweden is decentralised and mainly tax funded.^21^ The responsibility for healthcare is shared between the government, regions and municipalities. Regions are local authorities responsible for the provision of healthcare to the populations they serve and oversee both psychiatric inpatient and outpatient care. While Swedish psychiatric care is governed by rigorous standards of evidence-based practice, there are no formally established evidence-based requirements concerning nursing care on the wards, either with respect to the content or the structure of daily activities.

TT was introduced by the researchers to three different clinics across two regions in northern Sweden.^22^ The three clinics decided to implement TT at one ward each. Two of these wards cared for patients with substance use disorders, while one focused on patients with affective and anxiety disorders. Assistant nurses, registered nurses (RNs), consultants and ward managers are generally employed at these wards. The wards were tasked with appointing implementation leads who were trained during a 1 day workshop led by the research team. There was a clear agreement that the wards owned the implementation while the research team was involved in following the process through data collection and supporting the implementation leads.^10^

### Participants

As this study focused on organisational and leadership perspectives related to implementation, participants were limited to mid-level leaders and implementation leads involved in introducing TT at the wards. All mid-level leaders and implementation leads who took part in implementing TT at the three wards were invited to participate in this study. Mid-level leaders managed the participating wards while implementation leads were staff members with key responsibility for introducing and supporting the implementation of TT. Six months after TT had been implemented at these wards, mid-level leaders and implementation leads were asked to take part in follow-up interviews. In total, five mid-level leaders and seven implementation leads were involved in implementing TT. All 12 were invited to participate in this study. One of the implementation leads declined participation.

The 11 participants who agreed to participate were aged between 31 years and 53 years (median 45 years). Five of the participants were male and six were female. The implementation leads who agreed to participate in this study consisted of one assistant nurse, one occupational therapist and four RNs. Three of the RNs had advanced-level training in psychiatric and mental health nursing.

### Interview guide and data collection

Data were collected during November and December 2017 by author JM who is a female associate professor and senior researcher in psychiatric nursing and has extensive training and experience in interviewing and qualitative research. The interviewer did not have an established relationship with the participants before the interviews. Participants were informed about the interviewer, her credentials and her role in introducing TT and researching the implementation. Data were collected through semistructured interviews. To capture relevant aspects of implementation, the interview guide was developed using the CFIR framework.^17^ Involving mid-level leaders and implementation leads, it was deemed appropriate to ask questions about the ‘Innovation’, ‘Inner setting’ and ‘Individuals’ domains. Please see online supplemental appendix A for an English translation of the interview guide. The interviews began with an open question: ‘How did you experience starting with TT?’. Other examples of questions were ‘How do you experience TT compared with the previous work on the ward?’, ‘What resources are needed to start working with TT?’ and ‘What kind of training do you think is needed to start working with TT?’.

Eleven participants were interviewed in seven interviews. A combination of four individual interviews, two dyadic interviews and one group interview (with three participants) were conducted for logistical reasons, as some participants were unable to be scheduled for individual interviews due to time constraint or being unable to travel for the interviews. Participants were interviewed at their place of work, and no one except the participants and interviewer were present during the interviews. Notes were made during interviews to follow up on the answers given by the participants. The interviews were recorded and transcribed verbatim and lasted between 37 min and 52 min (median 41 min). No repeat interviews were carried out, and transcripts were not returned to participants for comments. Since all eligible participants were invited to participate, no further sampling was undertaken, and data saturation was not used to guide the sample size.

### Analysis

The content of the interviews was analysed deductively by author AG and guided by the steps described by Kyngäs and Kaakinen.^19^ A structured analysis matrix based on the domains and constructs of CFIR was created.^23^ The interviews were read several times in order to become immersed in the data and get a sense of the whole. Units of analysis were identified and evaluated for correspondence with the aim of the study. All units of analysis relevant to the aim were recorded in the analysis matrix, with no instances of data relevant to the aim falling outside its structure. Data were coded by AG, and the analysis was continually discussed among the authors. Due to the extensiveness of CFIR, there were instances when codes seemed to fit with more than one construct. In these cases, the authors re-examined the construct descriptions to make sure the code was assigned to the proper construct. The analytical findings were not returned to participants for feedback.

### Ethical considerations

The participants received written and verbal information about the study, and those who chose to participate provided written informed consent. To protect the participants, each interview transcript was pseudonymised, and the results are presented on a group level. Since there might have been a risk that the participants would feel scrutinised due to the subject of the interviews, there was an emphasis on conveying information that the study aims to explore the implementation of TT and not the individual’s performance.

A study protocol has been published.^22^

## Results

The deductive content analysis resulted in 15 categories sorted into 11 constructs and four domains of CFIR. The result is presented in table 1.

**Table 1:** CFIR domains, constructs and abstracted categories

Category	Construct	Domain
Addresses a lack of care content	Relative advantage	Innovation
Staffing and scheduling demands Organisational disruptions	Structural characteristics: work infrastructure	Inner setting
Reinforcing meaningful care	Mission alignment	
Moderate funding requirements Inadequate premises	Available resources: funding and space	
Ensuring increased understanding	Access to knowledge and information	
Living up to, and being, a leader	Mid-level leaders	Individuals: capability, opportunity and motivation
Being a persistent motivator Management support and motivation	Implementation leads	
Lack of knowledge Building consensus	Innovation deliverers	
Formal and informal follow-ups	Teaming	Implementation process
Setting reasonable goals	Planning	
Adjustability of approach	Tailoring strategies	

CFIR, Consolidated Framework for Implementation Research.

### Innovation

According to CFIR, this domain is about the characteristics of the innovation itself, in this case TT. The participants talked about TT from the perspective of the relative advantage of TT as an innovation compared with current practice.

#### Relative advantage

The participants perceived TT as more advantageous than the current practice at the wards where TT was implemented and addressed a lack of care content at the wards. The structure of the innovation increased the opportunity to spend more time with patients and provided them with new information about the patients.

##### Addresses a lack of care content

While the participants did not specifically address other innovations at their workplaces, they did discuss how TT was beneficial compared with how care was being delivered before TT was introduced. Further, participants described the need for having an innovation that increased the opportunity for nursing staff to spend time in activities together with patients.

The participants talked about how they felt positive towards TT when they were first introduced to it, and they felt it was an innovation that could structure work in a way that allowed them to spend more time with patients. They described how being with the patients sometimes took a back seat to administrative duties and that TT was able to free up and structure the day so that time spent with patients would be guaranteed. Implementation was facilitated as TT also aligned with what they wanted to do together with patients compared with what they did now.

We discussed the content as well… What should we do with our patients except give them pills, so we thought that this was worth trying. (Participant 11)

Participants discussed how little was offered for patients in inpatient care apart from medication or watching TV. They also described how TT would benefit them in exploring how patients acted in certain situations and how this would be important information to communicate with, for instance, consultants.

### Inner setting

This domain is about the setting where TT was implemented and about both general characteristics of the setting as well as characteristics more specific to implementing TT in that setting. In a wider sense, the inner setting domain can, according to CFIR, have multiple levels and may include hospitals and schools as well as individual units, classrooms, etc.

#### Structural characteristics: work infrastructure

This construct centres around how tasks and responsibilities are organised and how organisation and staffing levels can support the inner settings. This included how staffing could be a major barrier to implementation but how thorough planning alleviated this concern. Disruptions in the organisation, such as reorganisations and summer vacations also affected the implementation and delivery of TT.

##### Staffing and scheduling demands

Participants described how staffing concerns were a major barrier to implementing and sustaining TT in several ways. Initially, participants described concerns that a lack of available nursing staff as well as nursing staff scheduling issues would make TT challenging to implement. However, thorough planning regarding what time TT would be scheduled proved to be a facilitating factor in implementing TT. The participants highlighted how the staff’s schedule had to be taken into consideration when deciding on what time of day TT was planned. Doing TT when there was plenty of staff available helped the implementation. Other considerations regarding staffing also had to do with how the nursing staff would be available to assist consultants when they needed them throughout the day.

The consultants continued working with the patients then, and they need a surrounding organisation that works. Like all the time. So there was a lot of that, how are we going to solve that if the staff is completely released. Certain hours. (Participant 8)

##### Organisational disruptions

The lack of nursing staff and consultants was also particularly evident during summer, when many of the regular staff were on vacation, and the ward had temporary nursing staff working. Participants expressed that summers were difficult to manage overall, and this had an impact on being able to do TT. Other events, such as reorganisations and merging of different wards also had an impact on the ability of the ward to fully implement and deliver TT.

Yes, sometimes, based on the care situation and how things looked during certain periods, with merged wards and care demands being so high, it was sometimes not possible to have TT. (Participant 6)

Some participants referred to these events as ‘wartime psychiatry’ as the resources were stretched thin and only the absolute necessities were carried out.

### Mission alignment

This construct is about how implementation of TT aligned with the perceived mission of their care, which included being close to and spending time with patients. This alignment was a facilitator for implementation of TT.

#### Reinforcing meaningful care

Participants described how TT was perceived as being in line with what they felt was their job in the first place, which also facilitated the implementation of TT. Participants also described how closeness to the patients was their main priority, as opposed to doing administration, and that spending time with the patients was something they missed doing. They also described how creating a relationship with the patients was crucial and an important instrument in psychiatric care and that TT aligned with this mission. This extended to the rest of the staff as well, and they showed interest in the intervention due to this.

Both this desire from us in the staff, and that we want to be involved with the patients, that’s why we work with healthcare. It’s not just for administrating, running around in corridors, but finding ways to work close, or being close to the patients and have this opportunity for dialogue. (Participant 6)

This alignment with the perceived mission of psychiatric care also facilitated the delivery of TT. Participants said that implementing TT was something they could do and which they felt improved the care for the patients. This also helped participants realise that there was more that could be done with the patients and that it facilitated a better connection with the patients. The participants highlighted how they felt that TT was like going back to a previous form of psychiatry where the relationship itself was vital and healing.

### Available resources: funding and space

This construct is about funding and space as factors related to resources in the setting and how they affect implementation efforts. While funding requirements were moderate and not an issue for the participants, the facilities were considered inadequate and dedicated space for joint activities would have been preferable.

#### Moderate funding requirements

Participants described how TT was facilitated as reasonable funding was available for implementing and delivering TT. When TT was introduced to the participating wards, some funds were available from the research team for purchasing equipment and this was considered helpful and a good way to get TT off the ground. Even so, participants did not consider funding a barrier when TT was implemented as the costs were considered moderate and that purchasing the needed equipment was something that could have been done whether TT had been implemented or not. Mid-level leaders did not feel the need to clear these sorts of costs with higher-level management when they considered that spending these funds improved the content of care.

It’s been clear, for maybe 15 years, that managers very clearly know what their budget is like and we talk a lot about budgets and always have, even with the staff. And that some things are perfectly fine. (Participant 6)

Participants compared the cost of the material and equipment needed with what they thought the cost of ‘as-needed medication’ would be, and that they thought the funding needed for delivering TT was low when compared with this form of medication.

#### Inadequate premises

A perceived barrier to implementing and delivering TT was the availability of suitable spaces and facilities at the wards. The participants highlighted that the space available when delivering TT was inadequate and that they would have wanted a space more suited for activities. One of the barriers associated with this was that the available space at the ward was small and that it felt crowded when the staff and patients occupied it for different activities. Another barrier to delivering TT was that the crowded spaces made the ward feel loud and messy when a mix of both calmer and more physical activities took place at the same time.

The table tennis table, it got pretty crowded when it was there, and then, if someone wanted to sit and do something calmer, the space is very small and it can become messy very fast. (Participant 7)

The participants pointed out that in some instances the space they had available to them also served as the dining hall and that a specific room just for activities would have been desirable. One effect of sharing the space for TT with the dining hall was that all traces of the activities had to be removed for the space to function as a dining hall afterwards, which was perhaps not a major obstacle but nevertheless a barrier to delivering TT.

### Access to knowledge and information

This construct relates to the access of training and information needed to successfully implement TT. The initial information about TT was considered adequate but certain information such as the intervention rationale could have been made clearer for nursing staff, which could have increased the understanding of TT.

#### Ensuring increased understanding

The participants underlined the need for the implementation deliverers to understand the purpose of TT from the outset. While they did feel that the information they received at the beginning of the process was adequate, some participants also stated that they wished for clearer information about which days and what time of day was suitable for delivering TT. They stated that the purpose and rationale of the intervention in turn could have been further clarified for the nursing staff and that this might have led to improved engagement in the intervention and further facilitated the implementation process.

Or maybe clarify the purpose? That there is a clear rationale about why you do certain things. What you think that you want to affect with what you do. (Participant 9)

Participants highlighted the training and knowledge level in more general terms. They expressed that new and inexperienced staff in particular needed more knowledge and training about patients’ needs in order to better motivate patients to take part in activities. This could include training in conversational and communication methods.

### Individuals: capability, opportunity and motivation

In this category the participants described barriers and facilitators related to the individuals and their roles in connection with the implementation of TT. This structure was chosen in order to closely follow the CFIR structure as well as to differentiate between mid-level leaders, implementation leads and innovation deliverers.

#### Mid-level leaders

In this construct, participants who were mid-level leaders discussed their role in the implementation process and how capability, opportunity and motivation played a part in their efforts to be leaders facilitating the implementation of TT.

##### Living up to, and being, a leader

Participants described the mid-level leaders’ *capability* to live up to the role of leaders and the knowledge and skills needed to facilitate the implementation process. One of the skills that participants discussed was the ability to motivate the staff and keep the interest for the intervention going at the ward as well as aid in making it part of routine. This could include taking part in the intervention together with the staff and motivating the staff to deliver the intervention even on days when it seemed more difficult or when there was a staff shortage.

Now we have started this, even though we may be short of staff today, we’re going to do this anyway, because it, you have seen before what a positive effect it has. (Participant 4)

Another aspect of the skills needed by mid-level leaders included the ability to communicate to other agents, such as consultants, other wards and outpatient clinics. This communication could be about the intervention and how staff and patients would be mostly unavailable during the time of TT, and that certain tasks would have to be scheduled during other times of the day.

Mid-level leaders also needed to have an *opportunity* to be leaders in the setting in order to facilitate the implementation. The participants highlighted how mid-level leaders had to spend their time elsewhere and not on improving the content of care and this in turn affected the ward. The lack of leaders’ presence in the day-to-day work presented a barrier to implementing TT.

Mid-level leaders’ *motivation* could be either a barrier or a facilitator in the implementation process. Mid-level leaders themselves expressed that they needed to be engaged in and see the importance of the innovation for the implementation to work. They expressed that it could be easy for the staff to get wrapped up in other tasks at the ward and that they had to be motivated to actively help the staff remember to focus on the intervention. This motivation came from their own conviction that the intervention was important for the ward.

### Implementation leads

Participants who were implementation leads talked about how their capability to motivate the staff was crucial for delivering TT but also that, similarly to the mid-level leaders, they needed to feel supported in their work by their managers.

#### Being a persistent motivator

The implementation leads, similarly to the mid-level leaders, underlined being able to motivate the staff as an important *capability*. This could happen during several occasions throughout the process, both when introducing TT to the rest of the staff, but also during the process of delivering TT. This included conveying a positive attitude regarding the intervention and about the research conducted during the implementation process rather than demonstrating an ambivalence or insecurity about the process.

The implementation leads considered themselves as vital in maintaining the intervention. They expressed that they were needed to get the intervention going and making sure that other staff members did not prioritise other tasks when TT was scheduled. If they were not present, there was a risk that the intervention wasn’t delivered on that day. On the other hand, when TT had been running for some time, the need for the implementation leads to be present was not as great as in the beginning.

It was a bit dependent on, whether it was the first time or the last, that you had this responsible person, someone who starts up and could steer when others started prioritising other things. (Participant 2)

Implementation leads who also introduced the intervention to the staff at the wards explained that they felt insecure in explaining the intervention and the rationale and that they worried that they would explain it incorrectly.

#### Management support and motivation

The implementation leads talked about barriers connected to the *opportunity* to act as implementation leads. Just like their mid-level leaders’ counterparts, implementation leads needed to feel support from management in that TT was part of the ward’s routine. They also expressed that they found it difficult to prioritise TT at times of greater workload.

The *motivation* for implementing TT could be a facilitating factor for implementation leads. Participants mentioned how engaging in TT together with patients during the implementation process meant that the time they spent was worth more than just that 1 hour. It could also lead to a more open connection with the patients which in turn could make it easier for both the patient and the carer to talk to each other. These perceived positive effects motivated the implementation leads to continue with the implementation process even if the workload was high and likened this to being a starter motor for the process. If they could sustain long enough, the process itself would eventually be self-sustained.

### Innovation deliverers

Some of the mid-level leaders and implementation leads also took part in delivering TT and spoke about the barriers and facilitators from that point of view, as well as from what they perceived about other innovation deliverers. This included noting innovation deliverers’ lack of knowledge in interacting with patients, as well as a need for consensus among the innovation deliverers.

#### Lack of knowledge

The participants described the deliverer’s *capabilities* as sometimes having a lack of knowledge of interacting with persons with mental ill-health and feeling comfortable with somatic issues but not equally so with psychiatric issues. They described attitudes that included expressing an opinion that if the ward was ‘too cosy’, the patients would not want to be discharged. Similarly, they described how perhaps a lack of knowledge or insecurity about how to interact with patients could lead to ridiculing the intervention. This could also mean feeling insecure about whether the patients would want to engage in TT or whether suitable activities could be identified, even if this did not turn out to be a major issue.

There were a lot of concerns from the staff that the patients wouldn’t want to or that they wouldn’t identify suitable activities, but it became pretty clear that for the most part you do identify activities. (Participant 9)

Similarly to what the participants described in the previous sections, having the *opportunity* to perform as innovation deliverers was dependent on staffing levels and the workload at the ward. When staffing was minimal and the workload high, doing TT was sometimes not prioritised. The participants specifically mentioned that they felt that the RNs were more stressed about TT than other staff, that it was yet another task that needed to get done each day.

#### Building consensus

Participants highlighted that getting everyone ‘on board’ was important for *motivation*. This meant having an attitude that it is possible to introduce change, having a will to do it and an interest in being with the patients. While not everyone had to be on board from the beginning, having almost everyone eventually moving in the same direction greatly facilitated the implementation.

I don’t think that everyone has to be on board, although maybe many people still need to be on board, or at least several people, and then you can bring more people along over time. (Participant 7)

This motivation could come from seeing the benefits of TT and feeling that they were part of the process and receiving feedback on their part. Participants said implementation was more successful when deliverers felt their experience and knowledge were valued, rather than just following orders from managers.

### Implementation process

The participants described barriers and facilitators related to the process of implementation of TT in terms of activities and strategies.

#### Teaming

Participants described how they teamed up to implement TT on their wards. Teaming could mean both formal and informal meetings about TT and how to implement and deliver it in their settings. Having meetings which included mid-level leaders and implementation leads together with innovation deliverers decreased the risk of misunderstandings and facilitated the implementation.

##### Formal and informal follow-ups

Some participants said the lack of planning formal meetings before implementation was a barrier. They felt it would have been helpful to dedicate a half day to planning how TT should be introduced in their specific settings. Others described having different groups discussing aspects of both the implementation and the intervention before starting up. This helped them feel more prepared on the day they introduced TT.

We also started up groups on how to make sure this worked, so that when it did start up, we already had pretty good preparations regarding what we were going to do. (Participant 4)

Having team meetings with mid-level leaders and implementation leads together with the deliverers increased the spread of information about the implementation process and TT and decreased the risk of misunderstandings. At some wards, TT did not resume after periods of increased workload or periods where the regular staff were absent. The participants highlighted the need for teaming up again to restart the intervention. Participants described how there had been informal conversations about the positive effects of TT but that a more formal staff meeting was needed to get the ball rolling again.

### Planning

An important part about planning the implementation included setting reasonable goals and being prepared for the initial delivery of TT. This included not being too optimistic about which activities were possible and also improving planning about how information was communicated to the patients.

#### Setting reasonable goals

The participants described how they set goals and plans for the implementation. Part of this was being prepared for when TT was first delivered, such as having all the material ready in advance and that the staff was well informed. However, they also acknowledged that in some cases they set their goals too high compared with what was feasible, specifically regarding what activities were possible within TT. This meant that a focus should have been on freeing up time for TT rather than being too concerned with what activities were planned during TT.

Participants described setting goals regarding which time of day they planned on delivering TT and how to free up the staff for delivering TT. Nevertheless, participants found that some things could have been planned differently, specifically regarding how information about the activities was communicated to the patients. Others described not having talked much about the formal structure of delivering TT, and that this was something that could have been improved on.

If you want to decrease stress and possible negative emotions regarding the project when you start it up, then you have to sit down and look at the planning a bit more carefully than we did. (Participant 8)

Thorough planning for delivering the intervention included identifying who might be affected by TT, so that, for instance, consultants were informed that seeing patients was ideally scheduled for other times than during TT. Similarly, who were responsible for delivering TT each day was important, particularly during the initial start-up of TT.

### Tailoring strategies

Participants expressed the need of tailoring their strategies when implementing TT, specifically regarding how TT was implemented in their specific settings and being ready for unplanned events happening during the delivery of TT.

#### Adjustability of approach

Participants talked about the need to tailor how TT was implemented at their wards. This could mean adjusting how certain activities were presented to the patients and whether they would sign up for certain activities in advance or not. It also meant being prepared for unplanned events that might happen during TT. This could include having to admit new patients from the psychiatric emergency ward, adjust for patients that needed continuous monitoring or meeting with consultants that might visit the ward.

Another important way that the implementation of TT could be tailored to the specific settings was deciding on the time of day that was most suitable for delivering TT. This could even include adjusting someone’s schedule to better fit when TT was delivered.

Maybe someone can start at 1 pm, or that you have a schedule depending on the staffing situation. That’s something we must look at. Or what the staffing situation is like during the day. (Participant 5)

Tailoring the implementation strategy could also mean that at times the offered activity might not be something that all patients wanted to engage in, but it was better than not delivering TT at all that day.

## Discussion

Participants described TT as advantageous compared with the usual care provided at the wards, prompting them to reflect on what they wanted to offer patients beyond medication. This perceived advantage also underscored what participants felt was lacking in the care provided—specifically, time spent together with patients. However, the capability and motivation of the innovation deliverers, as described by implementation leads and mid-level leaders, suggest that not everyone agreed on TT’s advantages, with some even ridiculing it. This contradiction may indicate a divide among staff regarding what the content of care in PIC should be. Previous research has shown that TT and its core component—joint activities—can mean different things to different people. For instance, while joint activities can mean learning about oneself and the other, nursing staff also recognise that some colleagues perceive such activities as not being ‘real work’ or lacking intrinsic value.^24^ Other studies highlight similar differences in opinion regarding TT’s value and suggest that these discrepancies contribute to the burden of delivering the intervention.^12^

This divergence of opinion ties back to differing understandings of what the content of care in PIC should be. While academic studies highlight the importance of relational care with activities and interactions,^624^,^26^ this perspective is not always shared by all clinical staff members. Implementing an intervention like TT can both reinforce and challenge established conceptions of what constitutes meaningful care in PIC, making its implementation as divisive as the idea of engaging in joint activities itself. Achieving a broader, national consensus on what the content of meaningful care should be could facilitate the implementation of interventions focused on engagement in activities. However, the absence of a shared understanding or vision of nursing and care in PIC reflects both a limited and a more comprehensive understanding, potentially influenced by differences in education.^27^ This could also be reflected in a lack of guidelines regarding content of care, or that guidelines aren’t widely spread and known among nursing staff.

Closely connected to this is the finding pointing to the need for greater knowledge and a clear rationale for implementing TT, as reflected in the construct ‘Access to Knowledge & Information’. Additionally, the perceived capability deficiencies described in the ‘Innovation Deliverers’ construct further underscores this need. The results suggest training is essential for understanding, implementing and delivering TT, but so is a deeper appreciation of the importance of relationship building, joint activities and psychiatric nursing as a whole. This would suggest that training should include more than just building skills for delivering TT—it would benefit from also informing values and perspectives regarding the content of meaningful care.

Another way of viewing the issue of values, beliefs and attitudes is to consider how different understandings of meaningful and humane psychiatric care may influence staff engagement with joint activities. As highlighted in previous research, patients in PIC have described care as lacking empathy, communication and meaningful relationships, while boredom remains a common experience. TT is grounded in a view of nursing as relational and interactive, in line with Peplau’s^28^ interpersonal relationship theory, as well as with the Tidal Model, developed by Barker and Buchanan-Barker.^29^ Furthermore, in TT, meaningful activities are a way to encourage interpersonal relationships and address boredom. If staff members do not value joint activities as ‘real work’, this may influence how staff perceive the legitimacy and relevance of TT.

Mid-level leaders and implementation leads describe how they need to be leaders and motivators for the rest of the staff for them to perform as innovation deliverers. This includes having the skills necessary as well as being supported by management in leading the implementation effort. Being leaders and motivators while implementing TT, in combination with also facing obstacles in terms of inadequate training and having to promote shifts in attitudes, can be a significant challenge. Implementing interventions such as TT may therefore involve more than introducing new routines and practices, as it may also require reflection on existing values and beliefs related to psychiatric and mental health nursing and meaningful care. Previous research has also shown that experienced nurse leaders in psychiatric and mental health emphasise the importance of being inspirational and motivational in order to get people moving in the right direction.^30^ On the other hand, nurse leaders can experience powerlessness, frustration and irritation when the staff is unmotivated or have negative attitudes towards their tasks.^31^ While leaders in PIC have a responsibility to motivate staff, there is an even greater systematic or perhaps societal task in addressing some PIC staff members’ attitudes and values towards what constitutes meaningful care in PIC. More knowledge about the innovation deliverer perspective would be helpful to understand how their values and beliefs align or misalign with the leaders’ perspectives.

### Methodological considerations

This study was guided by CFIR both during the design of the interview guide as well as during the deductive content analysis. This contributed to a cohesive structure throughout the study. Kyngäs and Kaakinen^19^ mention that deductive content analysis relies on the same kinds of data sources as inductive content analysis but that the researchers will use prior knowledge to develop the structure for the data collection. A key reason for selecting CFIR for this project was the comprehensiveness of the framework. CFIR covers a multitude of determinants in a structured fashion, drawing from the implementation research literature. This provides an extensive set of determinant domains, subdomains and constructs. However, due to the breadth of CFIR, it was a complex task to select which domains to include in the interview guide, and several topics brought up by participants ended up relating to constructs not initially targeted by the guide. This made the interview data richer than originally anticipated. Rather than focusing solely on the interview data that matched the original constructs, and risk abandoning potentially valuable insights, the decision was made to code the entire interviews irrespective of which constructs the codes belonged to. Several units of analysis were therefore coded in constructs that were not originally part of the interview guide; however this contributed to a richer result, underlining important determinants as discussed by the participants. While Kyngäs and Kaakinen^19^ note that prior knowledge informs the structure in the deductive content analysis, they do not specify to what extent this should shape data collection instruments. Thus, although the interview guide was selective, the comprehensive coding ensured that the analysis captured relevant insights.

Data collection included individual dyadic and group interviews for practical reasons such as participant availability. Different interview formats may influence the data, such as individual interviews allowing for more personal reflections, while dyadic or group interviews may encourage shared reflections and discussions among participants. Morgan *et al*^32^ highlight the practical advantages of combining individual and dyadic interviews, but that interview questions need to be suitable for either situation. In this study, the interview guide was not created specifically for a certain number of participants and hence would most likely not have affected the data generated. As the interview questions were focused on implementation experiences in a professional context, the different interview formats were considered appropriate for the study aim. However, we acknowledge that participants may have expressed themselves differently depending on the interview format.

During the analysis process, no data were identified which didn’t match with any of the constructs of CFIR. However, there was some ambiguity regarding the coding of certain units of analysis with some overlap between constructs. This is an issue that has been highlighted by the authors of CFIR^18^ as some constructs are not always easily distinguishable from one another in certain circumstances. In this case, such uncertainties could be resolved by going back to the context of the units of analysis. However, we acknowledge that some findings could be viewed from multiple angles and readers may find that different constructs may be more fitting to their own context. This experience highlights the need for reflexivity and transparency when using CFIR deductively in qualitative research. While the comprehensiveness of the framework enabled a broad exploration of implementation determinants, researchers may need to carefully consider how constructs are defined and distinguished during analysis.

While all units of analysis could be coded to matching constructs, there were, however, several constructs that did not have any matching data. In particular, no units of analysis were coded to the constructs in the Outer Setting domain. The interview guide had no specific questions based on the constructs of this domain, but it would not have been surprising for the participants to discuss barriers and facilitators related to the Outer Setting domain, just like they had with other domains and constructs not covered by the interview guide. While we can only speculate about the reasons for this domain not being covered in the conversations, perhaps some of the constructs were not seen as obviously influencing the implementation effort from the participants’ point of view. Our experiences underline the importance of considering domain and construct selection during interview guide development and data collection, as selective use of constructs may influence which determinants are foregrounded in the analysis.

## Conclusions

The aim of this study was to explore mid-level leaders’ and implementation leads’ perspectives on implementing TT in PIC, analysed through CFIR. Key findings point to a discrepancy in the values and attitudes held by leaders and innovation deliverers regarding the content of meaningful care in PIC. Implementation is also impacted by challenges in being a leader and motivator for others. Taken together, this leads to a challenge for leaders in implementing interventions focused on joint activities in PIC. Similarly, there can also be hurdles with regard to staffing and the physical environment. This study’s focus was on leaders’ perspective on implementing TT in PIC and while not every aspect of implementation determinants was covered, an additional important area of research should be the innovation deliverers’ perspectives on implementing TT, as highlighted by the findings. In summary, implementation of TT in PIC faces barriers that may not be solely addressable through implementation and intervention training. Rather, the values and beliefs of intervention deliverers should also be taken into consideration when planning for implementing TT in this setting. By applying CFIR as an analytical lens, this study extends previous research on TT by identifying implementation determinants related to the intervention itself, organisational conditions, leadership perspectives, and individual understandings of psychiatric and mental health nursing.

## Supplementary material

10.1136/bmjopen-2026-117894 online supplemental file 1

## Data Availability

Data are available upon reasonable request.
