# Supplementary material for: Leader perspectives on implementing Time Together: a qualitative process evaluation using the Consolidated Framework for Implementation Research
Source: BMJ Open. 2026 Jun 24;16(6):e117894. doi: 10.1136/bmjopen-2026-117894 (PMC13295753; doi:10.1136/bmjopen-2026-117894)
Supplement: online supplemental file 1 [file bmjopen-16-6-s001.docx]

# Appendix A

## Interview guide and demographics questions

| **Introduction** |
| --- |

1. How did you experience the initial implementation of TTT? In what way, and why?
2. What motivated you to start using TT? What did the decision process look like?

| **Characteristics of individuals** |
| --- |

**Knowledge and beliefs**

1. How did you feel about starting with TT? Why?

**Self-efficacy**

1. How confident did you feel in your ability to initiate TT before you actually did it? Why?

| **Intervention characteristics** |
| --- |

**Relative advantage**

1. How do you perceive TT compared to your previous work on the ward?
   1. What does it add?
   2. What benefits do you experience?
   3. What disadvantages do you experience?
   4. What consequences did it have?

**Adaptability**

1. What changes do you believe are needed to make it easier to initiate TT?

| **Inner setting** |
| --- |

**Available resources**

1. What resources are needed to initiate TT? Which are most important?
   1. Have you received or would you like to receive any other resources? If yes, which ones?
   2. Which resources have you been lacking?

**Access to information and knowledge**

1. What kind of training do you think is needed to initiate TT? For you? For others in your situation?
   1. What kind of further education or professional development is needed?
2. What happened to TT after we left on May 31st?
3. Have you continued working with TT on the ward?
   1. How did you come to that decision? What was the reasoning? Who was involved? How did you decide who should be included in the decision-making process?

| **Closing** |
| --- |

1. Is there anything else you would like to add or share about your experience with TT?

| A | What is your age? | ____years |
| --- | --- | --- |
| B | What is your gender? | - Male - Female - Other: |
| C | What is your profession? | - Occupational Therapist - Social Worker - Registered Nurse - Nurse Specialist - Enlisted Nurse - Other: |
