# Supplementary material for: Leader perspectives on implementing Time Together: a qualitative process evaluation using the Consolidated Framework for Implementation Research
Source: BMJ Open. 2026 Jun 24;16(6):e117894. doi: 10.1136/bmjopen-2026-117894 (PMC13295753; doi:10.1136/bmjopen-2026-117894)
Supplement: Supplementary file 2 [file bmjopen-2026-117894.reviewer_comments.pdf]

PEER REVIEW HISTORY

BMJ Open publishes all reviews undertaken for accepted manuscripts. Reviewers are asked to complete a checklist review form and are provided with free text boxes to elaborate on their assessment. These free text comments are reproduced below.

ARTICLE DETAILS

Title (Provisional)

Leader perspectives on implementing Time Together - a qualitative process evaluation using the Consolidated Framework for Implementation Research

Authors

Glantz, Andreas; Lindgren, Britt-Marie; Nilsson, Ingeborg; Westerlund, Anna; Graneheim, Ulla Hällgren; Molin, Jenny

---

VERSION 1 - REVIEW

**Reviewer**	**1**
**Name**	**Faro, Elissa**
**Affiliation**	**Hackensack Meridian Hackensack University Medical Center, Center for Discovery and Innovation**
**Date**	**15-Apr-2026**
**COI**	**None**

Thank you for the opportunity to review this manuscript, which uses CFIR to structure the evaluation of implementation of Time Together intervention in psychiatric inpatient care settings. The manuscript provides an interesting perspective on implementation in a specific context; there are some considerations that would help the reader understand the findings more cohesively and better situate the contribution into the larger field and help with overall clarity.

Some suggestions that might help strengthen the argument overall and clarify the contribution:

• to better distinguish the research amongst the extensive qualitative literature on Time Together, especially given that it is being delivered in the context for which it was developed. While CFIR is an important theoretical frame for this study, perhaps the introduction and discussion/conclusion could focus more on what is different with CFIR as the analytic lens.

• Perhaps the above can include a rationale for why patients weren't included in the study.

• to include more direct quotation data in the results and less summarizing; it will perhaps make this application of the CFIR domains and constructs more distinct and clear.

• It may be that the inclusion of implementation outcome data might be outside the scope of this manuscript, but it would be interesting to know if implementation varied across the three sites and relatedly, but also independently, if responses differed between the sites

• The one sentence descriptions of the constructs feel redundant; If the construct is Mission Alignment; a sentence that says this construct is about how implementation aligns with the mission feels less helpful. Perhaps the introduction to each construct could provide an overview of the concepts within it or some other explanation of how it was identified or coded or some other contribution.

• The structure of Individuals section is unclear; why organize it by role when the findings are relatively similar across constructs and domains. If this is important, then it should be clarified why this organization was chosen.

• The argument in the discussion that individuals' beliefs and values should be de-implemented doesn't really make sense, as these are usually core to an individual's identity and likely outside the scope of de-implementation. The authors could clarify that perhaps individuals' beliefs and values need to be identified and considered when implementing and might result in different implementation strategies or something like that, but I don't think beliefs and values can be de-implemented.

**Reviewer**	**2**
**Name**	**Pelto-Piri, Veikko**
**Affiliation**	**Orebro universitet**
**Date**	**16-Apr-2026**
**COI**	**None**

To the Editor and Authors

Thank you for the opportunity to review this interesting and relevant manuscript. It makes a valuable contribution to the literature on implementing evidence-based interventions in psychiatric inpatient care. I have several suggestions regarding both style and substance. The revisions I consider necessary are: 1) clarification of methodological aspects in the methods section, 2) a more substantive discussion of the practical application of CFIR in implementation, and 3) if the de-implementation of values, beliefs and attitudes is to remain in the discussion, this argument requires grounding in appropriate references from medical ethics.

Abstract

Review the abstract, for example, The intervention Time Together is explained twice and the main findings are not presented enough.

Background

The manuscript currently opens with a focus on the Swedish context. It would strengthen the paper considerably if the opening sentences addressed why this study is relevant to an international readership. You could start by addressing the ethical dimensions of inpatient psychiatric care first, drawing on either a human rights or professional ethics perspective and connect it Time Together. This would provide an important foundation for the rest of the paper.

The introduction is brief and would benefit from expansion in the following areas:

- The intervention. Time Together is currently described in two separate places, briefly in the background and more fully in the methods section. It would be preferable to provide a thorough description in the background and remove it from the methods entirely.

- CFIR. The framework needs a clearer and more detailed introduction. Even as a reader familiar with CFIR, I found myself needing to look up additional information to fully follow the manuscript. A well-designed table summarising the key domains and constructs would be a practical solution.

- Rationale. The rationale for conducting this study is currently underdeveloped. There is a gap in the literature regarding leadership perspectives on implementing this type of intervention in psychiatric settings, and this should be stated clearly and compellingly. The aim needs to be better justified.

Aim

I would suggest reframing the aim to foreground the leadership perspective rather than the framework, for example: "The aim of this study is to explore mid-level leaders' and implementation leads' perspectives on implementing Time Together in psychiatric inpatient care, analysed through the Consolidated Framework for Implementation Research."

This is of course only a suggestion, but I note that the manuscript itself places considerably more emphasis on the leaders than on CFIR as such.

Methods

The methods section contains a degree of redundancy. For instance, lines 49–50 could be removed.

The study design section could be substantially shortened, participant selection, for example, does not belong there.

I would suggest that one of the senior authors reviews the manuscript and remove unnecessary wording and proofreading throughout, for example "a smorgasbord of determinant domains" might be replaced by “an extensive set of determinant domains”?

The COREQ was used for quality, but the reader should not need to go to the protocol, important facts need to be in the manuscript.

As noted above, the Intervention subsection could be removed from the methods and expanded in the background instead.

Context is crucial for interpreting qualitative research. Given that the interview guide did not include questions about the outer setting, a strong contextual description is particularly important. The current description is commendable, but I would suggest adding two elements:

- Societal level. It should be made explicit that whilst Swedish psychiatric care is subject to high standards of evidence-based practice, there are no formal evidence-based requirements regarding nursing care or social interaction on the ward, neither in terms of the content of the day, nor in relation to preventing coercive measures, verbal and physical aggression, or other adverse incidents. This point is touched upon early in the manuscript but could be stated more clearly and placed under "Context".

- Clinic level. Is there an expectation from clinic management that Time Together will be implemented, or is this expectation held primarily by the research team? This is likely to have had implications for the leaders' experience of the implementation process and should be clarified.

I would suggest renaming "Data collection" to "Interview guide and data collection".

I would also welcome a brief discussion of the reasoning behind the construction of the interview guide. Given the breadth of CFIR, with its many constructs and sub-constructs, it is not possible to address all of them. What considerations guided the selection of constructs included in the guide?

The analysis section is too brief. Was all coding conducted by a single author? How was the quality of the coding assured? Did any co-author conduct independent coding for comparison, or review a portion of the coded material? Were there instances of uncertainty about which construct a unit of analysis should be assigned to, and if so, how were these resolved?

Table 1 is largely redundant and could be removed. If the authors feel it has value, providing two examples per construct and placing it as an additional file would be a reasonable alternative.

Discussion

A matter of style: lines 34–39 could be removed to allow the discussion to open directly with the main findings, which would improve clarity.

Regarding the methodological considerations section: despite a reasonably thorough discussion of CFIR-related methodology, the different interview formats used (individual, dyadic, and group) are not mentioned, nor is any consideration given to what these differences might mean for the data. This should be addressed.

I would also suggest including a section discussing the empirical application and practical use of CFIR, see for example

Safaeinili N, Brown-Johnson C, Shaw JG, Mahoney M, Winget M. CFIR simplified: Pragmatic application of and adaptations to the Consolidated Framework for Implementation Research (CFIR) for evaluation of a patient-centered care transformation within a learning health system. Learn Health Syst. 2019 Sep 26;4(1):e10201.

or similar publications. This could include a reflection on what the authors learned from using CFIR in this context and any practical guidance they might offer to other researchers considering the framework.

Finally, regarding de-implementation: as the authors themselves note, de-implementation is conventionally concerned with low-value care. I am not convinced that de-implementation is the appropriate conceptual lens for addressing values, beliefs and attitudes. A more productive approach might be to ground the paper's value base at the outset, since interventions such as Time Together rest on commitments to professional ethics and human rights and then engage with this discussion from that foundation. A relevant reference here may be Fulford, who argues that psychiatry must be an evidence-based and values-based practice, and/or, maybe using the concept of "cognitive dissonance".

VERSION 1 - AUTHOR RESPONSE

	Reviewer 1	
1	To better distinguish the research amongst the extensive qualitative literature on Time Together, especially given that it is being delivered in the context for which it was developed. While CFIR is an important theoretical frame for this study, perhaps the introduction and discussion/conclusion could focus more on what is different with CFIR as the analytic lens.	Thank you for your insightful comment. We have revised the background, discussion and conclusion to better reflect the novelty of this study compared to previous research on Time Together.
2	Perhaps the above can include a rationale for why patients weren't included in the study.	Thank you for this comment. We have added a clarifying sentence in the Methods section explaining why these participants were selected.

3	To include more direct quotation data in the results and less summarizing; it will perhaps make this application of the CFIR domains and constructs more distinct and clear.	Thank you for this comment. Additional participant quotations have been included to further illustrate key findings and clarify the application of CFIR domains and constructs. At the same time, the results have intentionally retained an analytical focus in line with the qualitative content analysis approach used in the study.
4	It may be that the inclusion of implementation outcome data might be outside the scope of this manuscript, but it would be interesting to know if implementation varied across the three sites and relatedly, but also independently, if responses differed between the sites	Thank you for this comment. We agree that examining variation in implementation and responses across sites would be of interest. However, the aim of this study was not to compare sites or assess outcomes, and the sample was not designed to support comparisons. A systematic comparison between sites would require a different study design and is therefore beyond the scope of this manuscript.
5	The one sentence descriptions of the constructs feel redundant; If the construct is Mission Alignment; a sentence that says this construct is about how implementation aligns with the mission feels less helpful. Perhaps the introduction to each construct could provide an overview of the concepts within it or some other explanation of how it was identified or coded or some other contribution.	Thank you for this insightful comment. We have now rewritten the construct descriptions to better reflect the concepts within the constructs.

6	The structure of Individuals section is unclear; why organize it by role when the findings are relatively similar across constructs and domains. If this is important, then it should be clarified why this organization was chosen.	Thank you. We have clarified this choice in the appropriate Results section.
7	The argument in the discussion that individuals' beliefs and values should be de-implemented doesn't really make sense, as these are usually core to an individual's identity and likely outside the scope of de-implementation. The authors could clarify that perhaps individuals' beliefs and values need to be identified and considered when implementing and might result in different implementation strategies or something like that, but I don't think beliefs and values can be de-implemented.	Thank you. This concept has been removed from the Discussion section and elsewhere.
	Reviewer 2	
1	Review the abstract, for example, The intervention Time Together is explained twice and the main findings are not presented enough.	Thank you for this comment. We have reviewed the abstract and are hopeful that we have improved the abstract.
2	The manuscript currently opens with a focus on the Swedish context. It would strengthen the paper considerably if the opening sentences addressed why this study is relevant to an international readership. You could start by addressing the ethical dimensions of inpatient psychiatric care first, drawing on either a human rights or professional ethics perspective and connect it Time Together. This would provide an important foundation for the rest of the paper.	Thank you for this insightful comment. The beginning of the Background has been updated to reflect the relevancy for international readers.

3	The intervention. Time Together is currently described in two separate places, briefly in the background and more fully in the methods section. It would be preferable to provide a thorough description in the background and remove it from the methods entirely.	Thank you for your suggestion. The description of the intervention in the Methods section has been consolidated into the Background.
4	CFIR. The framework needs a clearer and more detailed introduction. Even as a reader familiar with CFIR, I found myself needing to look up additional information to fully follow the manuscript. A well-designed table summarising the key domains and constructs would be a practical solution.	Thank you for this comment. We carefully considered expanding the description of CFIR and adding an additional summary table. However, we assessed the current level of detail as appropriate for the scope of the manuscript, particularly as the manuscript already includes a table presenting the CFIR domains, constructs and resulting categories identified in the analysis, and each construct is also described when introduced in the Results section. Since the CFIR framework is highly comprehensive, a full summary table of domains and constructs would be extensive, and we therefore considered it more appropriate to refer readers interested in a more detailed overview to the original CFIR publications. Nevertheless, if the reviewer still finds aspects of the framework unclear, we would very much welcome more specific suggestions regarding which areas would benefit from further clarification.
5	Rationale. The rationale for conducting this study is	Thank you for this important comment. The rationale has

	currently underdeveloped. There is a gap in the literature regarding leadership perspectives on implementing this type of intervention in psychiatric settings, and this should be stated clearly and compellingly. The aim needs to be better justified.	now been rewritten and the aim justified to better reflect the literature gap and the focus on leadership perspectives.
6	I would suggest reframing the aim to foreground the leadership perspective rather than the framework, for example: "The aim of this study is to explore mid-level leaders' and implementation leads' perspectives on implementing Time Together in psychiatric inpatient care, analysed through the Consolidated Framework for Implementation Research."<br>This is of course only a suggestion, but I note that the manuscript itself places considerably more emphasis on the leaders than on CFIR as such.	Thank you for this thoughtful suggestion. We have now updated the aim to better reflect the manuscript's emphasis on leadership.
7	The methods section contains a degree of redundancy. For instance, lines 49–50 could be removed.	Thank you for your comment. The Methods section has been thoroughly revised, including Study design and Participants, both with regards to redundancy and length.
8	The study design section could be substantially shortened, participant selection, for example, does not belong there.	Thank you for this comment. The Study Design section has been shortened and revised per your suggestion.
9	I would suggest that one of the senior authors reviews the manuscript and remove unnecessary wording and proofreading throughout, for example "a smorgasbord of determinant domains" might be	Thank you for your suggestion. All authors have reviewed the resubmission.

	replaced by "an extensive set of determinant domains"?	
10	The COREQ was used for quality, but the reader should not need to go to the protocol, important facts need to be in the manuscript.	Thank you. We have updated the manuscript and checklist to make sure these items are also present in the manuscript.
11	Context is crucial for interpreting qualitative research. Given that the interview guide did not include questions about the outer setting, a strong contextual description is particularly important. The current description is commendable, but I would suggest adding two elements:<br>- Societal level. It should be made explicit that whilst Swedish psychiatric care is subject to high standards of evidence-based practice, there are no formal evidence-based requirements regarding nursing care or social interaction on the ward, neither in terms of the content of the day, nor in relation to preventing coercive measures, verbal and physical aggression, or other adverse incidents. This point is touched upon early in the manuscript but could be stated more clearly and placed under "Context".<br>- Clinic level. Is there an expectation from clinic management that Time Together will be implemented, or is this expectation held primarily by the research team? This is likely to have had implications for the leaders' experience of the implementation process and should be clarified.	Thank you for your comment. We have updated the manuscript with more details on these topics.

12	I would suggest renaming "Data collection" to "Interview guide and data collection". I would also welcome a brief discussion of the reasoning behind the construction of the interview guide. Given the breadth of CFIR, with its many constructs and sub-constructs, it is not possible to address all of them. What considerations guided the selection of constructs included in the guide?	Thank you for your comment. We have updated the manuscript with details regarding the development of the interview guide.
13	The analysis section is too brief. Was all coding conducted by a single author? How was the quality of the coding assured? Did any co-author conduct independent coding for comparison, or review a portion of the coded material? Were there instances of uncertainty about which construct a unit of analysis should be assigned to, and if so, how were these resolved?	Thank you for your thorough comment. We have updated the analysis section and are hopeful that this made the analysis process clearer.
14	Table 1 is largely redundant and could be removed. If the authors feel it has value, providing two examples per construct and placing it as an additional file would be a reasonable alternative	Thank you for this suggestion. We have removed Table 1.
15	A matter of style: lines 34–39 could be removed to allow the discussion to open directly with the main findings, which would improve clarity.	Thank you. Lines 34-39 has now been removed.
16	Regarding the methodological considerations section: despite a reasonably thorough discussion of CFIR-related methodology, the different interview formats used (individual, dyadic, and group) are not mentioned, nor is any	Thank you for your comment. We have revised the Methodological considerations to include considerations about the interview formats.

	consideration given to what these differences might mean for the data. This should be addressed.	
17	I would also suggest including a section discussing the empirical application and practical use of CFIR, see for example Safaeinili N, Brown-Johnson C, Shaw JG, Mahoney M, Winget M. CFIR simplified: Pragmatic application of and adaptations to the Consolidated Framework for Implementation Research (CFIR) for evaluation of a patient-centered care transformation within a learning health system. Learn Health Syst. 2019 Sep 26;4(1):e10201. or similar publications. This could include a reflection on what the authors learned from using CFIR in this context and any practical guidance they might offer to other researchers considering the framework.	Thank you for this important suggestion. The discussion section has been revised to reflect our experiences of using CFIR in this context.
18	Finally, regarding de-implementation: as the authors themselves note, de-implementation is conventionally concerned with low-value care. I am not convinced that de-implementation is the appropriate conceptual lens for addressing values, beliefs and attitudes. A more productive approach might be to ground the paper's value base at the outset, since interventions such as Time Together rest on commitments to professional ethics and human rights and then engage with this discussion from that foundation. A relevant reference	Thank you for this comment. We realise that the use of de-implementation in this case is unconventional and the discussion section and other sections have been revised to remove this concept.

	here may be Fulford, who argues that psychiatry must be an evidence-based and values-based practice, and/or, maybe using the concept of "cognitive dissonance".	

VERSION 2 - REVIEW

Reviewer **1**

Name **Faro, Elissa**

Affiliation **Hackensack Meridian Hackensack University Medical Center, Center for Discovery and Innovation**

Date **02-Jun-2026**

COI

Thank you for the opportunity to review the revised manuscript. The co-authors have thoughtfully and comprehensively addressed the reviewers' comments and the manuscript is much more robust methodologically and the clarity is improved. I might just suggest that while the findings are not-generalizable, the authors could consider a sentence in the conclusion regarding how other researchers might use this research.

Reviewer **2**

Name **Pelto-Piri, Veikko**

Affiliation **Orebro universitet**

Date **26-May-2026**

COI

The authors have addressed the issues I raised in an adequate manner
